# A qualitative exploration of service user views about using digital health interventions for self-management in severe mental health problems

**DOI:** 10.1186/s12888-018-1979-1

**Published:** 2019-01-21

**Authors:** Natalie Berry, Fiona Lobban, Sandra Bucci

**Affiliations:** 1 Division of Psychology and Mental Health, Medicine and Health, Manchester Academic Health Science Centre, School of Health Sciences, Faculty of Biology University of Manchester, Brunswick Street, Manchester, UK; 2 Spectrum Centre for Mental Health Research, Division of Health Research, School of Health and Medicine, Lancaster University, Lancaster, UK; 3 Greater Manchester Mental Health NHS Foundation Trust, Manchester, UK

**Keywords:** Digital health, mHealth, eHealth, Psychosis, Bipolar disorder, Qualitative

## Abstract

**Background:**

The development of digital health interventions (DHIs) for severe mental health problems is fast-paced. Researchers are beginning to consult service users to inform DHIs; however, much of this involvement has been limited to feedback on specific interventions post-DHI development. This study had two aims: 1. explore service user views towards DHIs for severe mental health problems; and 2. make recommendations for specific content within DHIs based on service user needs and suggestions.

**Methods:**

Qualitative interviews with eighteen people with severe mental health problems focussed on two domains: 1) views about DHIs for severe mental health problems; and 2) ideas for future DHI content and design features. Data were analysed thematically.

**Results:**

Participants responses were captured in five key themes: 1) DHIs could be empowering tools that instigate reflection and change; 2) society is already divided; DHIs will further increase this divide; 3) considerations must be made about who has access to DHI data and how this data may be used; 4) DHIs should not be delivered without other support options; and 5) DHIs should provide a positive, fun, practical and interactive method for self-management.

**Conclusions:**

Participants found DHIs acceptable due to the empowering nature of self-management and ability to take ownership of their own healthcare needs. However, concerns included the potential for digital exclusion, privacy and confidentiality and fears about DHIs being used to replace other mental health services. Service users want tools to help them self-manage their mental health, but also provide positive and recovery-focussed content that can be used in conjunction with other support options.

**Electronic supplementary material:**

The online version of this article (10.1186/s12888-018-1979-1) contains supplementary material, which is available to authorized users.

## Background

Evidence-based psychological interventions are recommended for people who experience severe mental health problems such as psychosis and bipolar disorder [1, 2]. However, factors including the costs of face-to-face therapy, lack of trained staff, and time and caseload pressures mean that timely access to support is not always available [3, 4]. Additionally, individuals experiencing severe mental health problems are often given little choice over the treatment options they receive [5–8]. Recent qualitative and survey-based studies with service users and health care staff have highlighted a number of factors that are barriers to the shared decision-making process, including perceptions regarding service user capability and capacity, aetiological beliefs, current risk to self and others, quality of research in the field, treatment costs and inadequate consultation times [9, 10]. Such barriers exist despite evidence showing that shared decision-making and increased treatment choice is desired by both service users and health care staff alike [6, 7, 10, 11] and can be beneficial for treatment-related empowerment [12] and personal recovery [13].

Alternative and complementary delivery options utilising digital technologies may improve access to psychological support options due to their widespread availability, reduced reliance of direct input from clinicians and potential to empower individuals with treatment choice and control [14]. To this end, the development and evaluation of digital tools for mental health is a priority in the UK National Health Service (NHS) Five Year Forward View for Mental Health [15] and researchers are currently exploring the potential for digital technologies to deliver self-guided interventions and assessments for severe mental health problems, with promising effects. For example, researchers have found correlations between assessments of psychotic and mood symptoms delivered via smartphone apps and in traditional gold-standard formats [16–18]. Additionally, preliminary evidence suggests small improvements in: quality of life and recovery [19]; community functioning [20]; negative symptoms, general psychotic symptoms and mood [21]; positive symptoms, excitement, general psychopathology and disorganisation [22]; and wellbeing [23] after the delivery of DHIs. However, further large-scale studies exploring the specific mechanisms that may elicit change are warranted [20]. Evidence for the feasibility, acceptability and potential effectiveness of DHIs is rapidly progressing [24–26], with some evidence of service user consultation in the development process (e.g. [8, 27–30]). Furthermore, recent survey-based studies have reported that many people with severe mental health problems are amenable to receiving technology-delivered support [31–33] and already use technology to facilitate self-management and assessment through activities such as information-seeking, symptom monitoring, medication and appointment reminders and receiving support from others online [31, 34, 35]. However, evidence regarding service user views towards DHIs and ideas for future developments is limited and survey-based designs have not gathered in-depth and detailed information that can be yielded qualitatively [36].

Whilst DHIs for individuals experiencing stress, depression and anxiety are readily available via self-help services [37] and the NHS apps library [38], DHIs for severe mental health problems are not yet widespread in their evidence base and availability. To create DHIs that are meaningful, acceptable and likely to be adopted by service users, there is the need to qualitatively explore end-users perspectives about how digital tools can be best used to deliver timely mental healthcare [36, 39]. There is a real danger of low levels of DHI uptake and engagement if potential facilitators and barriers are not identified prior to development and if end-users are not collaboratively involved in the design process. Therefore, this study had two main aims: 1) explore the perspectives of individuals with severe mental health problems towards self-guided DHIs (apps and websites) to identify potential facilitators and barriers to adoption; 2) make recommendations for design features and content within DHIs based on service user needs and suggestions.

## Methods

### Sampling and recruitment

Participants (*N* = 18) were purposively recruited from two mental health trusts by presenting the study at team meetings and asking staff to pass details of the study to clients. Additionally, mailing lists were used to invite people who had previously stated an interest in participating in studies. The recruitment materials contained the researcher’s contact details (office telephone and work email address) for individuals to contact directly, but they were also given the option to contact the researcher via a member of their care team if preferred. Participants were given at least 24 h after receiving the participant information sheet to decide whether they would like to take part. Reasons for not participating included: loss of contact (*n* = 9); no time to commit to participating (*n* = 2); not wanting to consent to providing a designated health care contact (*n* = 2); and no interest in talking about experiences (*n* = 1). Participants were aware from the participant information sheet that the lead researcher was a PhD student investigating individuals views towards the development of DHIs for people who experience severe mental health problems. The purposive sampling method was to ensure a representative number of participants with bipolar and schizophrenia-spectrum diagnoses. Eligibility criteria were: 1) diagnosis of a schizophrenia-spectrum disorder or bipolar disorder; 2) 18–65 years of age; 3) capacity to provide informed consent; 4) sufficient English language skills; and 5) Internet and mobile phone access. Recruitment stopped when data sufficiency was reached; that is, based on analysis of transcripts and discussion amongst the research team, it was agreed that no additional themes were generated from the data.

### Procedure

One-to-one interviews were conducted by the lead author either in-person at the participant’s own home (*n* = 7), in a health care setting (*n* = 2) or at the University of Manchester (*n* = 1) or via telephone (*n* = 8) between April and October 2016. Prior to each interview, participants gave written informed consent and completed a demographics and technology ownership questionnaire to contextualise the sample. Interviews were administered using a topic guide (Appendix A) developed for the study based on review of the literature and informed by Smith’s (1995) guidelines [40] and questioning was focused on participant perceptions of self-guided interventions delivered via websites and smartphone apps. The topic guide was pilot tested with lay volunteers (*n* = 2) and language refined based on these comments prior to interviewing participants. Interviews lasted between 25 and 82 min. Ethical approval was granted from Cambridge South Research Ethics Committee (Ref: 14/EE/0059). The lead author (female PhD psychology student) received formal institutional training in qualitative research methods, topic guide development, interview style and analysis and supervision from the two other members of the research team (experienced female academic consultant clinical psychologists).

### Data analysis

**Fig. 1:**
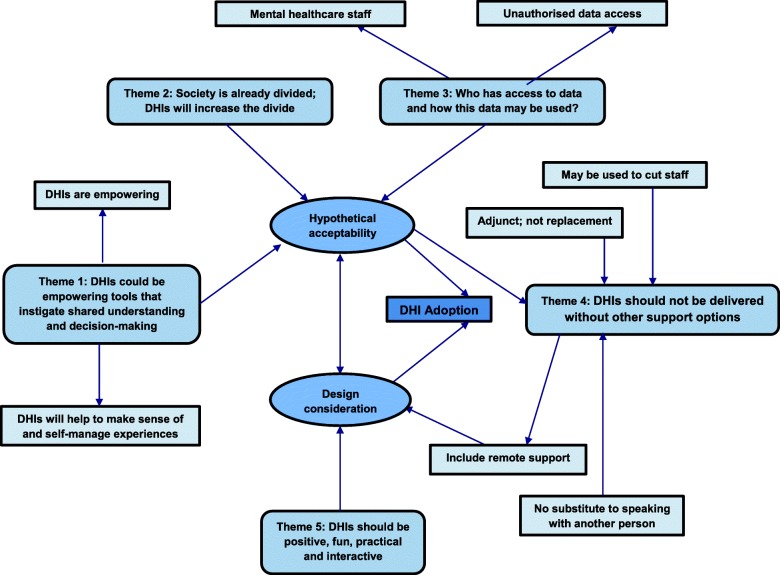
Thematic map

Interviews were audio-recorded, transcribed verbatim by the lead author and subject to thematic analysis, as described by Braun and Clarke (2006) [41]. First, the lead author read each transcript several times for data familiarisation. The two other members of the research team independently reviewed two transcripts and assigned descriptive codes to data relevant to the study aims. The research team then met to discuss and compare the codes for reliability and consensus. The lead author coded the remaining transcripts in a cyclical process, moving backwards and forwards between transcripts as new codes emerged. Once coding had been completed, related codes were arranged into candidate themes by clustering together and collapsing codes based on similarities and differences using NVivo version 10 software. The research team met again to review and refine the themes, which were discussed against the coded data and the final themes and subthemes were agreed. Although distinct, themes were also linked and are represented in the thematic map shown in Fig. 1.

### Reflexivity

The research team work on trials implementing DHIs for severe mental health problems; we therefore acknowledge that this may have influenced interviews and analysis. As such, several considerations were made to ensure transparency in the reporting process. A reflective journal was maintained throughout data collection and analysis to enhance the quality and credibility of the study and questions about views towards DHIs were phrased broadly to ensure participants were not primed to give certain views. At the beginning of each interview the researcher also reassured participants that all views given were valid and important, to prevent them from feeling they had to provide only positive views. There were no existing relationships between the research team and participants. The research team met regularly during analysis to discuss emerging themes and how beliefs and experiences may affect data analysis and interpretation. For example, the research team held in-depth discussions regarding biases associated with being actively involved in several DHIs currently under investigation and were careful to ensure that negative or conflicting views towards DHIs were fairly represented in the analysis.

## Results

As shown in Table 1, participants’ age ranged from 25 to 63 years (*M* = 37.3, *SD* = 11.5). The majority of the sample was female (*n* = 11; 61%), had a diagnosis of schizophrenia (*n* = 7; 39%), were unemployed (*n* = 12; 67%) and were in current contact with mental health services (*n* = 17; 94%).

**Table 1:** Participant demographics and technology ownership

Demographic Information	Frequency	Percentage
Gender		
Male	7	38.9
Female	11	61.1
Ethnicity		
White British	15	83.3
British Asian	2	11.1
Black British	1	5.6
Employment: ^a^		
Working full-time	2	11.1
Working part-time	2	11.1
Self-employed	1	5.6
Student	1	5.6
Unemployed	12	66.7
Highest level of education: ^a^		
High school	2	11.1
College/sixth form	7	38.9
Some university	1	5.6
University (degree awarded)	3	16.7
University (postgraduate degree awarded)	5	27.8
Diagnosis: ^a^		
Schizophrenia	7	38.9
Schizoaffective disorder	1	5.6
Bipolar disorder Type I	5	27.8
Bipolar disorder Type II	2	11.1
Bipolar disorder NOS	3	16.7
Previously received psychotherapy?		
Yes	17	94.4
No	1	5.6
Currently receiving psychotherapy?		
Yes	4	22.2
No	14	77.8
Technology Ownership	Frequency	Percentage
Smartphone ownership?		
Yes	16	88.9
No	2	11.1
Social media profile?		
Yes	16	88.9
No	2	11.1
Tablet computer ownership?		
Yes	12	66.7
No	6	33.3

^a^Due to rounding, percentages may not add up exactly to 100%

Five themes were identified: 1) DHIs could be empowering tools that instigate reflection, understanding and change; 2) society is already divided; DHIs will increase this divide further; 3) considerations must be made about who has access to DHI data and how the data may be used; 4) DHIs should not be delivered without other support options; 5) DHIs should provide a positive, fun, practical and interactive method for self-management. Themes are elaborated below and evidenced by key quotations embedded within the text.

### Theme 1: DHIs could be empowering tools that instigate shared understanding and decision-making

Participants often noted that support from staff was constrained by barriers such as working hours, childcare provisions and anxieties about leaving the house (*n* = 14). As such, DHIs were viewed as a method to give individuals the control and means to access self-management options when they were needed at any time:

*“…they [DHIs] could be very liberating… it could be like having your mental health support going alongside”* (Participant 8: female; 59; Bipolar disorder).

Some participants (*n* = 3) also commented that DHIs could allow them to start and stop “sessions” whenever they wished, further enhancing control. Occasions where it had been difficult to remember and explain feelings and experiences to friends, family and staff were also described by several participants. In this situation, DHIs could be used to record feelings, experiences, and could facilitate conversations by showing records to others. This information-sharing was viewed as empowering, because it placed individuals in the expert role and allowed them to take ownership of information disclosures:

*“I think just that sense of ownership and having control I guess of it; feeling like it’s your decision and you’re… more involved in managing that process…”* (Participant 11: male; 36; bipolar disorder).

Therefore, DHIs were referred to as “liberating” because of the potential to allow service users to take control of their own mental health and recovery through ease and choice of access, readdressing the power imbalance between service users, clinicians and family members.

The need for a clearer understanding of their own symptoms and experiences was expressed by participants (*n* = 10). DHIs were viewed as a method to facilitate understanding through clear and accessible information about topics such as symptoms, early warning signs and diagnosis. Many participants (*n* = 13) also viewed symptom monitoring as a method that could help them understand triggers and patterns, which could be used to elicit behaviour change; this was particularly true for participants who experienced bipolar disorder:

*“Just being able to track your mood… over certain periods of the day on a regular basis and just to be able to record simple things alongside those that would then help you to identify if there were any particular cycles…”* (Participant 11: male; 36; bipolar disorder).

However, others (*n* = 3) felt they would not personally benefit from DHIs because they were already able to recognise triggers and patterns and regularly used coping strategies that were helpful for them. Participants living in supported accommodation in particular believed they already received adequate support:

*“I… see the doctors all the time and they keep an eye on me so… I wouldn't need to use it or find anything out”* (Participant 2: male; 37; schizophrenia).

Whilst some participants stated they were already self-aware, they believed that these tools would be useful for people who were experiencing symptoms for the first time or recently diagnosed, had just experienced relapse or had recently been discharged from inpatient settings (*n* = 2):

*“… it would be extremely helpful for people who are newly ill or haven’t experienced something like what they’re experiencing before… I’ve been ill for so long that I’ve sort of experienced everything already”* (Participant 10: female; 31; bipolar disorder).

There was the overall view that DHIs could help people contextualise and understand their experiences in a way that was meaningful for them; although, the people who would benefit the most may be those experiencing symptoms for the first time.

### Theme 2: Society is already divided; DHIs will increase this divide further

There was the perception by almost all participants (*n* = 16) that DHIs would marginalise members of the community who did not have access to, or the ability to use, technology due to much needed services and support options becoming inaccessible and unavailable for these individuals:

*“[DHIs would be] great for people who've got it, the technology, but I think that's an equality issue… and there's so much dividing of people in this country going on at the moment… that would make the people who can't even more isolated”* (Participant 8: female; 59; bipolar disorder).

Devices and data costs were viewed as expensive and even cheaper or second-hand models often had poor battery life and storage. Indeed, one participant described being unable to download apps because of a lack of storage available:

*“…this phone’s not that good cos all the apps have got bigger than the memory. It’s a year and a half [old] now… so it won’t let me download anything”* (Participant 1: male; 25; schizoaffective disorder)

Indeed, the reflective journal maintained by the researcher highlighted the common theme that participants felt DHIs would not be accessible to all due to the digital divide. However, there was the caveat that participants did have technology access:

*“A striking viewpoint that is coming out from the interviews is the concern that some people will not have the technology ownership and skills needed to access DHIs. This was despite participants being active technology users who self-reported high levels of comfort in using technology in the demographic questionnaire, which suggests that participants were raising this concern on behalf of others”* (Interviewer, reflexive diary, after Participant 18).

The perception noted in the reflexive diary is exemplified by the view from a 25-year-old participant that older individuals would not have the technology skills required to engage with DHIs:

*“say someone who’s like 40 and they’re not used to, they don’t know how to use a computer… young people these days who know how to use all that technology, but older people won’t.”* (Participant 3: male; 25; schizophrenia)

Participants detailed potential strategies to overcome this issue, including installing computers and tablets in community settings, funding DHIs through the NHS, providing digital devices at discounted rates and technology skills training (*n* = 10). Participants felt DHIs should be simple and user-friendly and that information and instructions should be presented clearly using different mediums (*n* = 14). This would improve ease of use for people who may struggle to understand written information.

There was also the perception that people spend too much time using technology, which has contributed to poor communication and divides in society (*n* = 7). Lack of face-to-face communication within DHIs was viewed as being particularly isolating for people with poor social skills or those who are unable to see others regularly:

*“…you need this face-to-face contact otherwise people can stay in their bedrooms or in their living room and never leave because they're in this internet virtual world”* (Participant 9: female; 63; bipolar disorder).

Although there were real fears that DHIs could be inaccessible and may lead to isolation, some feasible suggestions were proposed to overcome these potential barriers to DHI adoption.

### Theme 3: Considerations must be made about who has access to DHI data and how it is used

Concerns were raised that DHIs, particularly those including symptom monitoring, could lead to people catastrophising and over-analysing feelings without the input of staff (*n* = 3). Additionally, some participants felt they would be unable to interpret the data without their care team (*n* = 2). Therefore, many participants wanted staff to have access to their data to aid diagnosis, explore further questions and facilitate shared decision-making (*n* = 15):

*“There's an outcome, somebody's bothering with it, somebody's looking at it, somebody's saying to you… how were you feeling a week ago, it looks as though you were low, what was happening in your life at that time”* (Participant 9: female; 63; bipolar disorder).

However, opinions were mixed with regards to how access should be granted. Many participants (*n* = 12) stated a preference towards taking the data to staff themselves during appointments to give them ownership over their data:

*“… you'd have to have the option of being able to choose, and not just like a one-off option at the start, but like with each graph or information…”* (Participant 5: female; 46; schizophrenia).

Two participants said they would prefer members of their care team to have automatic access for early warning sign detection and so staff could review information prior to appointments:

*“I just think it would be easier than you taking it in to them… and they’d have it there and then so they would tailor your appointment based on the information that they’ve got…”* (Participant 17: female; 29; schizophrenia).

Some participants (*n* = 4) also voiced concerns that data gathered from DHIs could be placed in their medical files without their knowledge and used as evidence for involuntary inpatient admissions, but at the same time, participants acknowledged that DHIs could prevent inpatient admission through early relapse identification. Therefore, different user needs led to mixed views regarding how mental health care staff should gain access to their data from DHIs.

Participants were uneasy about inputting personal information into DHIs due to concerns that individuals or organisations outside their care team might access their data (*n* = 7). For example, there were fears that the data could be acquired by the UK Department of Work and Pensions (DWP) and used to provide evidence for stopping disability payments:

*“…DWP will end up knowing oh this person's going through a good phase oh jolly good JSA down the job centre... we can be quite paranoid, but it's not paranoia when it really is happening” (*Participant 8: female; 59; bipolar disorder).

The potential to lose payments also raised concerns that people may feel pressured to be dishonest when completing assessments, which could inadvertently affect the mental healthcare they receive. Additionally, the potential for data being obtained by third party agencies (e.g. pharmaceutical companies) was also noted:

*“[an app] might have a tracking thing at the back to see… how often you're using it and what you're feeling, and then they'll try and sell you happy pills…”* (Participant 9: female; 63; bipolar disorder).

Participants (*n* = 5) spontaneously identified potential solutions to concerns expressed above; including terms and conditions associated with data sharing; consent processes; explicit statements that service users had been involved during DHI development; and that DHIs had been developed by a trusted source:

*“If it did have the NHS logo on then probably… I would feel more comfortable with it… you know if it was a recognised thing”* (Participant 5: female; 46; schizophrenia).

Therefore, whilst participants did express some fears regarding the use, privacy and confidentiality of their data, they would still engage with DHIs providing they were fully informed and reassured about the use of their data.

### Theme 4: DHIs should not be delivered without other support options

Participants (*n* = 15) feared that the development of DHIs would justify staff cuts in NHS mental health services and *“reduce access to other forms of therapy and support...”* (Participant 14: female; 34; bipolar disorder). These fears related to the perception that cuts were being made to NHS services and was reflected in the researcher’s journal at the time of interviews:

*“Participants are sometimes describing examples of cuts to services, reflecting on their own experiences and those of close family members or friends. In a time of austerity, it is likely that these views are contributing to concerns that DHIs would be used as an excuse to replace, rather than complement, face-to-face options”* (Interviewer, reflexive diary, after Participant 8).

Therefore, the provision of DHIs was viewed as an attractive option to the government that would lead to reductions in costs associated with trained staff. However, there was one participant who noted that the NHS needed to be more cost-efficient:

*“…how much [money] could that save the NHS… The mental health worker that comes around to see me once a fortnight is stretched so thin…”* (Participant 5: female; 46; schizophrenia).

Participants believed that the most important element of mental healthcare was being supported by another person. For this reason, participants (*n* = 16) were concerned that DHIs would not be as helpful or as effective as face-to-face interventions:

*“I just don't believe there's any replacement for the compassionate presence of another human being… I don't think technology can ever replace the soul, depth of connection and the enormous power that that can bring…”* (Participant 11: male; 36; bipolar disorder).

There were also situations where the only appropriate delivery method for psychological interventions particularly was in-person. For example, some participants felt psychoeducation or CBT-informed interventions could be successfully delivered digitally, but therapies such as person-centred, psychoanalysis or trauma-focussed CBT would not be helpful without the presence of a therapist. Some participants (*n* = 5) felt DHIs could be beneficial for people with low to moderate depression or anxiety, but not appropriate for those experiencing severe and complex problems because DHIs could not go as in-depth as face-to-face approaches:

*“If there was… a lower level disorder so brief periods of anxiety or very mild depression, then I would say maybe that would be something that would be beneficial”* (Participant 12: female; 34; bipolar disorder).

However, two participants stated a preference for DHIs, rather than receiving face-to-face support. Both participants explained that this preference was due to years of receiving different face-to-face support without benefit so believed that DHIs could be an acceptable and appropriate alternative:

*“It would be like having a little person at the side of you, a little friend… some app, that would do me…”* (Participant 5: female; 46; schizophrenia)

*“I’d prefer a mobile phone or a website… I generally find that people who go into mental health care and things only understand what they’re taught basically, which is the scientific view behind it and they’re not the kind of people who understand like that we’re spiritually different”* (Participant 16: male; 36; schizophrenia).

Participants also reflected on difficulties with being open about their experiences in face-to-face settings due to fears of being judged. DHIs were viewed by some as a potential avenue to address this issue by being faceless, thereby facilitating open and honest self-expression (*n* = 14):

*“I think it would just be easier to then open up to about how you’re feeling…when it’s between you and the application and it’s like you’re talking, but you don’t have to look at the person when you’re talking…”* (Participant 17: female; 29; schizophrenia).

Therefore, there was the almost universal belief held by the majority that DHIs could never substitute the warmth, empathy and human contact face-to-face support could provide, but may be useful for people experiencing mild to moderate symptoms or are uncomfortable openly discussing their experiences and feelings.

Although participants did not view DHIs as an alternative to face-to-face support, many (*n* = 17) could see DHIs working in conjunction with existing support options. For example, some participants suggested DHIs could be provided for people on waiting lists to help them understand their experiences, practice self-management strategies and identify needs and goals prior to therapy:

*“… to be able to turn up to your first appointment with… some information about yourself or with some charts or graphs… that brings some sense of meaning to the waiting time and gives a bit more context in that initial meeting”* (Participant 11: male; 36; bipolar disorder).

*“maybe if they, if there, is a long waiting list there could be some kind of technology prior to them getting face-to-face help… I’d definitely think there’s a place for it but then I don’t necessarily think it should be the be all and end all you know”* (Participant 13: female; 57; bipolar disorder).

Participants described experiences of staff asking them to record symptoms for use in appointments (*n* = 8). However, some detailed occasions where they had forgotten, or felt uncomfortable, completing paper-based reports so had retrospectively completed them. Participants felt using devices could help solve these problems:

*“Paper-based is a bit of a faff... I was always anxious that my family would find the mood diary…”* (Participant 10: female; 31; bipolar disorder).

DHIs were also viewed by three participants as a potential method to reduce number of sessions needed. For example, instead of seeing staff once a week, appointments could be arranged two weeks to monthly. Some participants said that it would be preferable if they could attend face-to-face appointments less frequently, but for a longer duration with a DHI to supplement:

*“...face-to-face for six weeks or online without the person there for a longer time… if I was going to get longer by it being online then I would favour that over just the face-to-face”* (Participant 15: female; 37; bipolar disorder).

There were some concerns that psychological interventions can raise particularly strong feelings, memories and questions for people (*n* = 4). Ordinarily, these could be managed by a therapist; however, a person may be left alone without answers or require support when using DHIs. Therefore, participants requested the inclusion of telephone, video chat, or instant messaging options should individuals need further support (*n* = 15). Contact details for support outside the DHI (e.g. charity helplines) were considered important in emergency situations. Social networking design features and moderated peer support forums from people with lived experience were viewed as valuable to help facilitate supportive discussions and shared understanding (*n* = 16):

*“… modules where you've got more forum interaction would be much easier to engage with and much more motivating”* (Participant 14: female; 34; bipolar disorder).

*“cos a lot of people that have mental health, that have schizophrenia, it gives them an option there that they can talk to people that go through the similar situations”* (Participant 4: male; 30; schizophrenia).

As such, the overall view expressed by participants was that DHIs could allow more people access to psychological interventions, not by replacing face-to-face support, but by using different methods of delivery together to improve care and extend choice.

### Theme 5: DHIs should provide positive, fun, practical and interactive methods for self-management

Participants noted that people often focus on the negatives of experiencing mental health problems. DHIs should challenge this negative stance and contain design features that provide positive, fun, practical and engaging activities to help foster self-management. A common suggestion (*n* = 12) for design features was stories written by people with severe mental health problems at different stages of their recovery, with a view to normalise and understand their experiences and provide hope for the future:

*“…you can read the medical part and the science part but that doesn’t touch your heart and that doesn’t motivate you… you need somebody’s experience to think well they’ve got that; they’ve got out the other end better”* (Participant 17: female; 29; schizophrenia).

Symptom monitoring was viewed as a potentially useful strategy to recognise triggers and patterns (*n* = 13); however, concerns were expressed that continually filling in information about symptoms could lead to ruminating and catastrophising (*n* = 3). Therefore, participants wanted monitoring to include positive feelings and activities, which could allow people to recognise the achievements and positive aspects of their lives. Participants also commented that information about mental health might not only be negative, but also boring and complicated. Gamification options were therefore viewed as fun and interactive, which could facilitate engagement (*n* = 5):

*“… cos if you was one of those people that weren’t motivated but the thing is like playing games… if you had a way to learn more about your illness and a game…”* (Participant 1: male; 25; schizoaffective disorder).

Participants strongly believed that DHIs should not focus entirely on mental health-related activities and, instead, incorporate exercises that were unrelated to mental health, but would still have a positive impact (*n* = 12). For example, during symptom emergence, some participants struggled with daily living skills such as cooking and managing their finances and productivity. Therefore, they recommended the inclusion of simple recipes, cleaning and gardening tips, craft ideas and money management advice. Four participants also described the positive impact of physical exercise on their mental health, but noted that it could be difficult to find the motivation to exercise or have the knowledge about the type of physical exercise to do:

*“I think it would be great to have something that says okay this is a low impact thing that you can do; this is a medium and a high and progress…”* (Participant 7: female; 29; bipolar disorder).

Difficulties with arranging face-to-face appointments due to anxiety were also raised and DHIs were viewed as a method to address these concerns. For example, appointment scheduling via DHIs was suggested as a design feature that would be a practical and useful tool for people to arrange appointments (*n* = 7). Additionally, some participants commented that it could be difficult to remember medication and appointments so felt DHIs could be a way to deliver reminders (*n* = 4). However, some felt there was not a need for reminders because they were able to remember, whilst others preferred traditional paper calendars.

Additional examples of information and activities participants wished to receive in DHIs included: 1) simple games to distract from thoughts and feelings; 2) lists of activities that take five minutes if people are at a loose end; 3) videos posted by others of outdoor activities that people can view if they are unable to leave the house; 4) music that may help improve mood or distract from thoughts or feelings; and 5) mindfulness and relaxation-based exercises. Therefore, participants wanted a variety of design features including mental health-related content such as symptom monitoring, reminders and scheduling, goal setting, coping strategies, peer support and psychoeducation, but also non-mental health recovery-focussed specific information and activities.

## Discussion

The aim of this study was to explore the perspectives of service users about the use of DHIs for severe mental health problems. Participants felt DHIs could be empowering through providing information and tools to give users choice and control over their recovery. However, potential barriers to adoption were also identified, including: the digital divide; fears about data protection and handling; and assumptions that DHIs would replace face-to-face care. A further aim of the study was to identify service user needs and recommendations to inform DHI design features. Whilst specific mental health-related features were suggested, participants also wanted non-mental health recovery-focussed content. Research has been limited to seeking service user views towards DHIs after receiving an intervention, rather than prior to intervention development. Therefore, this is one of the first studies, to our knowledge, to interview individuals with severe mental health problems to identify facilitators, barriers and recommendations for future DHI adoption within this population.

The control and autonomy over the type, time and location of intervention access was viewed as a benefit over traditional face-to-face care. People with severe mental health problems often describe feeling disempowered in healthcare choices and uninvolved in the decision-making process [42, 43]. Giving people choice over treatment options and promoting shared decision-making are priorities in the NHS Constitution Pledge [44] and targets for improvement by 2020 [45]. These findings indicate that the needs of service users and aims of service providers align. Therefore, to improve DHI acceptability and subsequent likelihood of adoption, DHIs should be created and positioned as avenues to empower individuals, give people choice and facilitate shared decision-making.

Participants were concerned that DHIs could create divisions in society through digital exclusion. These fears stemmed from cuts to disability benefits and the assumption that some people would lack the required technology skills. However, almost all participants reported smartphone ownership, Internet access and a high level of comfort with technology. Therefore, issues surrounding digital exclusion were not personally relevant to participants and assumptions about technology access may not be accurate. A meta-analysis of mobile phone ownership rates in people experiencing psychosis reported a narrowing gap in ownership in comparison with the general population [46]. However, some people remain digitally excluded [47] and participant concerns in the context of the current political environment must be considered as potential barriers to DHI adoption. Acceptable solutions were spontaneously divulged, including technology skills training, medical discounts and community setting access. Due consideration should be given to these issues when delivering DHIs.

Participants believed that entering data on a DHI in the absence of any clinician involvement may be meaningless. Therefore, participants were willing to grant the care team access to their data, but had mixed views about whether this access should be automatic or user-initiated.Traditionally, symptom monitoring approaches tend to involve the automatic transfer of symptom information [48]; however, these findings suggest service users should have control over information transfer, which echoes views expressed by staff [49] and individuals with bipolar disorder [50] and first episode psychosis [51]. Concerns over third party access were also expressed and may be a barrier to DHI uptake by people with severe mental health problems. Participants suggested the inclusion of terms and conditions regarding data use that are clearly presented and easy to understand, which contrasts with some mental health-related apps currently available for download [52].

The potential for the implementation of DHIs to be used as an ‘excuse’ to replace face-to-face care stemmed from observations of budget cuts to NHS services at the time of interviews. However, participants were positive about the provision of DHIs to enhance user choice, provide an adjunct to existing options and reduce the number of face-to-face appointments required. A recent analysis of user reviews of apps for bipolar disorder highlighted that users had reported using these apps in conjunction with face-to-face care [52], whilst qualitative studies with staff and people experiencing severe mental health problems stressed the need for continued support during DHI engagement [49, 51, 53–55]. Therefore, DHIs are seen by service users as a method to enhance face-to-face care, rather than replace existing support.

DHIs with social networking design features and moderated forums were frequently suggested. Peer support can provide hope for recovery and empowerment [56] and is recommended by NICE [1, 2]. Additionally, some individuals already report discussing their mental health online to receive support from others with lived experience [57]. Considerations such as forum moderation by trained peer supporters and specific structured topics were recommended by participants. Additionally, participants wanted remote support options attached to DHIs to allow users to ask questions and receive additional support if needed. Whilst there are limited studies that have compared the provision of remote support to no support, recent systematic reviews reported higher DHI acceptability and engagement when remote coaching and support were offered [36, 58]. These findings underline the potential for the incorporation of forums and remote support options within DHIs.

Participants wanted DHIs to contain interactive activities unrelated to mental health in addition to mental health-specific content. For example, some participants suggested gamification strategies, which have previously shown some promise in improving engagement with DHIs [59, 60]. The observed improvements in engagement and motivation to use DHIs in severe mental health problems warrants continued investigation. Importantly, participants wanted DHIs to include information and exercises not directly related to mental health, but that may still improve their quality of life. As such, it is important that DHIs not only focus specifically on symptoms, but also on recovery.

### Limitations

Participants were a small sample of individuals recruited through community mental healthcare teams mainly in the North West of England; all used mobile phones and had Internet access. Therefore, views expressed are unlikely to be representative of this population as a whole, but do highlight important considerations for DHI developers. Interviews were conducted mid- to late-2016. During this time, UK government-funded organisations were making well-reported service and funding adjustments to improve cost-efficiencies. Therefore, some views expressed by participants about DHIs directly related to their experiences of the political landscape at the time. Questions in the topic guide used in the qualitative interviews focussed specifically on self-guided DHIs that service users can use on their own or between therapy sessions, rather than interventions delivered during a therapy session. Interventions delivered via other technologies such as video-conferencing, telephone and virtual reality were, therefore, not considered. Additionally, researchers are beginning to explore how relapse can be predicted via passively collected smartphone data (digital phenotyping) [61]. Future research should explore both clinician and service user views towards passive data collection methods to determine its acceptability.

We purposively sampled participants based on diagnosis to ensure equal representation of severe mental health problems within the sample. However, we did not sample for other demographic or clinical characteristics such as age, education, ethnicity or current service access. We also set an upper-age limit of 65 due to community mental health teams at the time only open to individuals aged 65 or below. This limits the transferability of the findings beyond the group sampled. The time, resource and financial limits associated with the project also prevented the opportunity for participants to be re-interviewed or review transcripts or for respondent validation (member checking), whereby individual participants are re-contacted to provide their views on interpretation, analyses and drafts [62]. Although some researchers have highlighted potential pitfalls of respondent validation, including its time-intensive and potentially exploitative nature (see [63] for Discussion), employing this technique may have improved the validity of the findings. Finally, the researchers involved in the analysis are involved in DHI trials, which may have led to bias in interpretation. To mitigate this, regular research team meetings were scheduled to discuss potential biases and the first author kept a reflective journal during data collection and analysis.

## Conclusions

This study reports content and design features service users want to see included in DHIs for severe mental health problems and demonstrates the need for continued and improved service user involvement throughout all phases of the design process. The suggestions made by participants indicate that recovery-focussed and strengths-based content would be acceptable in addition to mental health-focussed content. Additionally, the findings demonstrate the need for continued consideration of the potential utility of symptom monitoring in clinical practice. Specifically, the automatic availability of symptom monitoring data for clinical use was perceived as advantageous to identify early warning signs and triggers and patterns and aid diagnosis. However, concerns were raised regarding third party access and the transfer of data directly to staff. Work is needed to understand how automatic transfer of data to services can still occur for those who want it, whilst maintaining their feelings of control and ownership. Due to the lack of qualitative research in the field, these findings are particularly novel and unique to the current study as they represent the service user’s voice about the role digital technology can play in mental health care. Finally, remote peer support design features were popular amongst participants, highlighting the need for continued implementation of social media and forum integration within DHIs.

## Additional file

Additional file 1: Qualitative topic guide used in qualitative interviews. (PDF 259 kb)

## Abbreviations

CBT: Cognitive Behaviour Therapy
DHIs: Digital health interventions
NHS: National Health Service
UK: United Kingdom
