# Supplementary material for: A qualitative exploration of service user views about using digital health interventions for self-management in severe mental health problems
Source: BMC Psychiatry. 2019 Jan 21;19:35. doi: 10.1186/s12888-018-1979-1 (PMC6341527; doi:10.1186/s12888-018-1979-1)
Supplement: Supplementary file 1 — Qualitative topic guide used in qualitative interviews. (PDF 259 kb) [file 12888_2018_1979_MOESM1_ESM.pdf]

The University of Manchester

Interest and use of technology for self-management purposes by individuals experiencing severe mental health problems: service user and clinician perspectives.

Interview guide: service users.

Version 2: 25/06/16

Principal Investigator

Natalie Berry (PhD student)
Room S42, Zochonis Building
School of Psychological Sciences
Brunswick Street
University of Manchester
Manchester
M13 9PL
Tel: 0161 306 0428
Email: natalie.berry@manchester.ac.uk

Supervisory Team

Dr Sandra Bucci, School of Psychological Sciences, University of Manchester.
Professor Fiona Lobban, Spectrum Centre for Mental Health Research, Lancaster University.

**Equipment:**	Participant Information Sheet<br>Consent forms<br>Encrypted audio-recorder<br>Demographics questionnaire

**Prior to interview:**	Participant Information Sheet – any questions?<br>Consent form – explain audio recording and limits of confidentiality.<br>Demographic data (separate sheet)

**Introduction:**	Introduce self, welcome, and thank participant for attending the interview. Ensure that the participant is comfortable.
**Consent:**	Re-confirm informed consent is still valid and participant still wishes to take part.
**Interview details:**	Outline interview procedures and expected length of time for the interview.<br>Remind the participant that the interview will be audio recorded, that participation is voluntary, that they have the right to withdraw at any point without penalty and offer pauses and breaks.
**Confidentiality:**	Explain confidentiality – everything the participant says during the interview will remain confidential; however, if the participant discloses a risk of harm to themselves or another person, the researcher will need to inform the named healthcare contact.
**Explain purpose of the study and take any questions:**	“Thanks again for meeting with me today. The interview is split into two parts. In the first half we will discuss what sorts of things you use the internet and your mobile phone for and any experiences you have using technology to support your mental health. In the second half we will move on to talk about your thoughts, views and ideas of people receiving mental health support online and through mobile phones. The interview will take around 45 minutes to complete, but for some

	people it may take a little bit more or less time. With your permission, the interview will be audio-recorded and then typed up to make sure we have an accurate summary of what you have said. Recordings will be securely stored in password protected computer files. Sometimes I may use quotes that you provide during the interview in publications, but I would always make sure that you would not be identifiable from these quotes. Do you have any questions at all?”
**Demographics questionnaire:**	Remind participant that the interview is not being recorded yet and ask participants to complete the demographics questionnaire.

Section 1: Current technology use to support mental health

**Domain**	
**General internet use:**	“My first question is just generally what sort of things do you use the internet for?”
**Information-seeking online:**	“Do you have any experiences of using the internet to find out information about mental health?”<br>***prompt****: what sort of information did you search for?*<br>***probe****: what did you think about the information that you found?*<br>***probe****: were there any websites that you found particularly helpful?*<br>***probe****: were there any websites that you found particularly unhelpful?*<br>***probe****: did you ever speak to a healthcare professional about any of the information you found online:*<br>***if yes*** *– how did you find that conversation went?*<br>***if no*** *– were there any reasons why you didn’t discuss the information you found online?*<br>***prompt****: what are the positive things about searching for information about mental health online?*<br>***prompt:*** *have you ever had any negative experiences when searching for information about mental health online?*

**Talking about mental health online:**	"Do you have any experiences of talking about your mental health on the internet?"<br>***prompt:*** *social media (e.g. Facebook, Twitter), blogs, forums*<br>***if yes*** - ***probe:*** *what sort of things have you said about your mental health?*<br>***probe:*** *why do write about your mental health online?*<br>***prompt:*** *can you tell me about any good things that have happened when you have spoken about your mental health online?*<br>***prompt:*** *has anything negative happened when you have spoken about your mental health online?*<br><br>***if no*** - ***probe:*** *what are your thoughts about using the internet to talk about mental health problems?*<br>***probe:*** *are there any reasons why you haven't spoken about your mental health online?*<br>***prompt:*** *what might be the benefits for people speaking about mental health online?*<br>***prompt:*** *do you have any concerns about people speaking about mental health online?*
**Other ways people use the internet for their mental health:**	"Are there any other ways you use the internet for your mental health?"
**General mobile phone use:**	"What sort of things do you use your mobile phone for?"
**Health smartphone applications:**	"Have you ever used any health-related smartphone applications?"<br>***prompt:*** *fitness apps, diet apps, smoking apps*<br>***if yes – probe:*** *how did you find using the app?*<br>***if no – probe:*** *are there any reasons why you haven't used any health smartphone applications?*

**Other ways people use mobile phones for their mental health:**	"Do you use your mobile phone to support your mental health?"<br>***prompt:*** *appointment reminders, medication reminders, smartphone applications*<br>***if yes -*** ***probe:*** *would you be able to tell me a bit more about that?*<br>***probe:*** *how was that helpful?*<br>***probe:*** *have you ever had any negative experiences when doing that?*<br>***if no*** - ***probe:*** *are there any reasons why you don't use your mobile phone for your mental health (e.g. appointment reminders, medication reminders, smartphone applications)?*

Section 2: Concept of technology-delivered interventions for SMI

**Introduce concept:**	"So we're now moving into the second part of the interview would you like to take a break at all or are you happy to continue? In this part we will be speaking about your thoughts, views and ideas about support options that could be offered on mobile phones applications or on websites. An example might be where people could be asked to put in how they're feeling on a website or mobile phone application and then they would receive coping strategies or hints and tips back based on what they've put in. Another example might be where people are offered information about mental health problems on a website or smartphone application and might have interactive exercises to complete that may help them cope with some of the things that they are experiencing. The important thing to remember is that this would be done just with a mobile phone or website without another person there."
**Domain**	

**Previous experiences of receiving therapy**	"Have you ever received therapy or counselling?"<br>***prompt:*** *give examples if unsure.*
**Support via a website**	"What are your thoughts about receiving a therapy using a website?"<br>***prompt:*** *"What might be the benefits for people receiving support through a website?"*<br>***prompt:*** *"What concerns would you have about people receiving support through a website?"*
**Support via a smartphone application:**	"What are your thoughts about receiving a therapy using a smartphone application?"<br>***prompt:*** *"What might be the benefits for people receiving support through a smartphone application?"*<br>***prompt:*** *"What concerns would you have about people receiving support through a smartphone application?"*
**Preferences for delivery method:**	"If you were offered a therapy in a face to face setting, a therapy delivered online, or a therapy delivered on a smartphone application, which would you be most likely to choose?"<br>***probe:*** *"Why would (insert preferred choice) be your preferred option?"*<br><br>"Which one would you be least likely to choose?"<br>***probe:*** *"Why would you be least likely to choose (insert least preferred choice)?*
**Overcoming access issues:**	"Not everyone is able to get access to the support that they need and there may be waiting lists to receive support. Do you think websites and mobile phone applications could overcome problems with access to help?"<br>***Probe:*** *"Why/why not?"*

**Privacy issues:**	"Do you have any concerns about the privacy or safety of support offered through mobile phone applications or the internet?"<br>***if yes* – *prompt:*** *"can you tell me a bit more about these concerns?"*<br>***if no* – *prompt:*** *"can you tell me a bit more about why you do not have any concerns about this?"*
**Willingness to receive support via technology:**	"If you were offered the option to receive a therapy on a smartphone application or on a website, would you want to receive it?"<br>***Probe:*** *"can you explain why/why not?"*
**Symptom monitoring:**	"There are now smartphone applications where you can receive alerts or prompts to complete questions on the application about your thoughts and feelings a few times day; for example your mood or anxiety, and you can then be sent graphs weekly, monthly and so on to show how you're feeling over time. What are your thoughts about this?"<br>***Prompt:*** *"What do you think might be the benefits of this type of application?"*<br>***Prompt:*** *"What concerns would you have about this type of application?"*<br>***Probe:*** *"How many times a day would you be willing to answer the questions?"*<br><br>"There has also been the suggestion that the information a person gives on this sort of smartphone application could then be sent to their healthcare professionals for example their psychiatrist or care coordinator for them to have to look over. What are your thoughts about this?"<br>***Prompt:*** *"What do you think might be the benefits of healthcare professionals getting this information?"*<br>***Prompt:*** *"What concerns would you have about healthcare professionals getting this information?"*<br>***Probe:*** *"Would you prefer that the healthcare professional*

	automatically receives this information or for you to take the information on the smartphone directly to them yourself? Why?"
**Healthcare professionals on social media:**	"Some people have suggested that social media accounts contain a lot of information about a person's daily life and could be used by a person's psychiatrist or care coordinator to see how their doing. What are your thoughts about healthcare professionals looking at clients social media accounts?"<br>***Prompt:*** *"What might be the benefits of healthcare professionals accessing this information?"*<br>***Prompt:*** *"What concerns would you have about healthcare professionals accessing this information?"*<br>***Probe:*** *"How would you feel about healthcare professionals you have had contact with accessing this information? Why?"*
**Researchers on social media:**	"What some researchers are now doing is automatically collecting mental health data for example tweets from people's social media accounts for research if the account is not set to private. What are your thoughts about researchers using data from peoples social media accounts for research"?<br>***Probe:*** *"Do you have any concerns that consent is not provided?"*
**Future ideas for technology-delivered interventions:**	"Can you think of anything you would like to see in future therapies offered online or through mobile phones?"<br>***If struggling:*** *"Imagine you were creating a mobile phone application or website for people experiencing (insert diagnosis) what sort of things would you like to see included that might be helpful?"*

Interview closedown

"Is there anything else that you would like to tell me that we haven't discussed, but you think might be relevant when thinking about mental health and the internet and mobile phones?"

“How have you found this interview today?”
“So I’ll also be interviewing other people about their experiences and thoughts about technology and mental health. How do you think this interview could be improved for future participants?”
“Ok I’ll now switch of the audio recorder.”

End of interview

Thank the participant for taking part.<br>Explain what will happen with the information provided.<br>Ask the participant whether they would like to receive a summary of the results.<br>Ask the participant whether they have any questions.<br>Offer a support phone call: “sometimes people take part in an interview and afterwards they have more questions to ask or they have been worrying about something they said. If you like, I can call you tomorrow just to check if any of this is happening for you – would you like me to do that?”
